# The Pasteur Methods

**Published:** 1887-01

**Authors:** 


The Pasteur Methods—Dr. Christopher Johnston read before the Baltimore Academy of Medicine a paper on his recent visit to Pasteur's dispensary and laboratory in Paris. Many foreign doctors were present at Pasteur's headquarters, eager observers, and there was also a numerous attendance of persons bitten by supposed rabid animals. Dr. Johnston said that recently bitten persons were inoculated with a thin pulp, made by crushing a portion of a dried spinal cord, ten days old, from a rabbit dead of rabies or hydrophobia. On the second day a ninth-day cord was employed, on the third day an eighth-day cord, and so on until the fresh cord of the preceding day was brought into requisition. Eighty-four patients were treated by the above method the day of Dr. Johnston's visit. But 351 persons were bitten last year in France by rabietic animals, yet M. Pasteur treated 1,700. The excess came from other countries. Rabbits die in six days when inoculated. They are inoculated from the brain of a rabbit which died of hydrophobia, while Pasteur's patients are inoculated from the lower part of the same rabbit's spinal column. As to the practical working of the system it was objected by Dr. Johnston that enough has not been done to demand the acceptance of Pasteur's theory. Time and the future labors of Pasteur and others must develop the truth that may reside in Pasteur's plan of treatment. Dr. Von Frisch, of Vienna, and Pasteur both fully inoculated animals, the former rabbits and the latter dogs, and then attempted their cure. All of Von Frisch's rabbits died, and Pasteur had only "a partial success." Pasteur objects to the slow method of Von Frisch, and urges rapid and repeated vaccinations, but his own success was but "partial".

---

